# 4-(4-Propoxybenzo­yloxy)benzoic acid

**DOI:** 10.1107/S1600536808016942

**Published:** 2008-06-13

**Authors:** Khushi Muhammad, M. Khawar Rauf, Masahiro Ebihara, Shahid Hameed

**Affiliations:** a Department of Chemistry, Quaid-i-Azam University, Islamabad 45320, Pakistan; b Department of Chemistry, Faculty of Engineering, Gifu University, Yanagido, Gifu 501-1193, Japan

## Abstract

The title compound, C_17_H_16_O_5_, is an important inter­mediate for the synthesis of side-chain ligands for polymeric liquid crystals. The prop­oxy and benzoic acid groups subtend dihedral angles of 4.36 (6) and 55.35 (6)°, respectively, with the central benzo­yloxy unit. The crystal structure is stabilized by an inter­molecular O—H⋯O hydrogen bond.

## Related literature

For related literature, see: Ahmad *et al.* (2003 ▶); Aranzazu *et al.* (2006 ▶); Cady *et al.* (2002 ▶); Hameed & Rama (2004 ▶); Hartung *et al.* (1997 ▶); Hussain *et al.* (2003 ▶, 2005 ▶); Kong & Tang (1998 ▶); Nazir *et al*. (2008*a* ▶,*b* ▶); Ribeiro *et al.* (2008 ▶); Shafiq *et al.* (2003 ▶, 2005 ▶); Wu & Hsu (2007 ▶); Wu & Lin (2007 ▶).
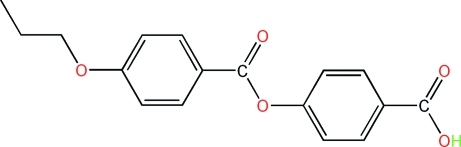


## Experimental

### 

#### Crystal data

C_17_H_16_O_5_
*M* *_r_* = 300.30
Monoclinic, 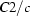

*a* = 21.063 (15) Å
*b* = 5.703 (4) Å
*c* = 24.437 (18) Å
β = 99.790 (9)°
*V* = 2893 (3) Å^3^
*Z* = 8
Mo *K*α radiation
μ = 0.10 mm^−1^
*T* = 123 (2) K
0.30 × 0.19 × 0.15 mm

#### Data collection

Rigaku/MSC Mercury CCD diffractometer
Absorption correction: empirical (*NUMABS*; Higashi, 1999 ▶) *T*_min_ = 0.970, *T*_max_ = 0.985
11426 measured reflections
3297 independent reflections
2824 reflections with *I* > 2σ(*I*)
*R*_int_ = 0.051

#### Refinement

*R*[*F* ^2^ > 2σ(*F* ^2^)] = 0.084
*wR*(*F* ^2^) = 0.146
*S* = 1.26
3297 reflections
201 parameters
H-atom parameters constrained
Δρ_max_ = 0.29 e Å^−3^
Δρ_min_ = −0.28 e Å^−3^

### 

Data collection: *CrystalClear* (Molecular Structure Corporation & Rigaku, 2001 ▶); cell refinement: *CrystalClear*; data reduction: *TEXSAN* (Molecular Structure Corporation & Rigaku, 2004 ▶); program(s) used to solve structure: *SIR97* (Altomare *et al.*, 1999 ▶); program(s) used to refine structure: *SHELXL97* (Sheldrick, 2008 ▶); molecular graphics: *ORTEPII* (Johnson, 1976 ▶); software used to prepare material for publication: *SHELXL97* and *TEXSAN*.

## Supplementary Material

Crystal structure: contains datablocks I, global. DOI: 10.1107/S1600536808016942/hg2407sup1.cif

Structure factors: contains datablocks I. DOI: 10.1107/S1600536808016942/hg2407Isup2.hkl

Additional supplementary materials:  crystallographic information; 3D view; checkCIF report

## Figures and Tables

**Table 1:** Hydrogen-bond geometry (Å, °)

*D*—H⋯*A*	*D*—H	H⋯*A*	*D*⋯*A*	*D*—H⋯*A*
O3—H3*O*⋯O4^i^	0.84	1.77	2.606 (3)	172

Symmetry code: (i) 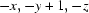.

**Fig. 1.**
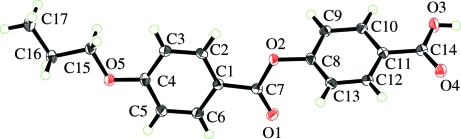
Molecular structure of (I) showing atom-labelling scheme and displacement ellipsoids at the 30% probability level.

**Fig. 2.**
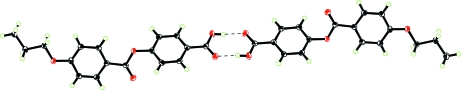
Showing hydrogen bonded molecules through N—H···O.

## supplementary crystallographic information

### Comment

Differently substituted aromatic carboxylic acids having benzene rings either joined directly through a covalent bond (Wu & Hsu 2007) or through some functional group, mostly an ester (Cady *et al.*, 2002; Wu & Lin 2007) or an olefin (Nazir *et al.*, 2008*a*; 2008*b*), have been investigated for their liquid crystal properties. Such acids have been used in the synthesis of intermediates for side-chain liquid crystal polymers (Kong & Tang 1998) as well as for main-chain liquid crystal polymers (Aranzazu *et al.*, 2006). In addition, the carboxylic acids, in general, have been used as intermediates in the synthesis of a large number of organic compounds (Hussain *et al.*, 2005; 2003; Shafiq *et al.*, 2005; 2003; Ahmad *et al.*, 2003). The pharmaceutical industry has also benefited from this class of compounds (Ribeiro *et al.*, 2008; Hameed & Rama, 2004). The title compound (I) was synthesized in our lab as an intermediate in the synthesis of side-chain liquid crystal polymers, by treating 4-hydroxybenzaldehyde with 4-propyloxybenzoylchloride followed by KMnO_4_ oxidation. In this report, the crystal structure of (I) is presented. Bond lengths and angles are within the normal ranges as given for benzoyloxybenzoic acids (Hartung *et al.*, 1997). The C(14)—O(4), C(14)—O(3),*C*(7)—O(1) and C(7)—O(2) bond lengths are 1.237 (3), 1.300 (3), 1.204 (3) and 1.367 (3) respectively, clearly indicating the partial double bond character of the carboxylate groups. The benzoic acid groups subtend dihedral angles [55.35 (6)°] with the central benzoyloxy moiety C(1)/C(2)/C(3)/C(4)/C(5)/C(6)/C(7)/O(1)/O(2). Two molecules related by an inversion center form a dimer *via* two hydrogen bonds composed of two carboxyl groups as shown in Fig. 2.

### Experimental

To a solution of 4-hydroxybenzaldehyde (0.032 moles) in 50 ml of triethylamine (TEA) was added an equivalent amount of 4-propoxybenzoylchloride with stirring and the mixture heated at 333 K for 1 hour. The excess TEA was removed in vacuo and the product, after recrystallization from hot ethanol, was subjected to KMnO_4_ oxidation. The 4-(4-propoxybenzoyloxy)benzaldehyde (0.025 moles) was dissolved in acetone (100 ml) and aqueous KMnO_4_ (0.025 moles) was added dropwise at room temperature with stirring. The stirring was continued for three hours when the reaction mixture was filtered and the filtrate acidified using 6M HCl. The product was purified by recrystallization from acetone. Yield: 93% (from 4-(4-propoxybenzoyloxy)benzaldehyde); m.p: 478-480.5K; IR (ν_max_, KBr, cm^-1^): 3100-2400, 1731, 1685, 1603, 1512, 1425, 1300, 1260, 1206, 1163, 1061, 1009, 758; ^1^H-NMR (300 MHz,DMSO-d_6_): δ  0.99 (3H, t, J = 7.2 Hz), 1.77 (2H, sex, J = 6.9 Hz), 4.05 (2H, t, J = 6.6 Hz),7.12 (2H, d, J = 8.7 Hz), 7.4 (2H, d, J = 8.7 Hz), 8.03 (2H, d, J = 8.7 Hz),8.08 (2H, d, J = 8.7 Hz), 13.02 (1H, bs); ^13^C-NMR (75 MHz, DMSO-d_6_): 10.75, 22.33, 69.91, 115.16, 120.85, 122.70, 128.79, 131.35, 132.60, 154.65, 163.82, 164.29, 167.12.

### Refinement

The O-bound H atom was refined isotropically. All the other H atoms were placed in idealized positions and treated as riding atoms, with C—H distance in the range 0.95–0.99 Å and *U*_iso_(H) = 1.2*U*_eq_(C) or 1.5*U*_eq_(C).

### Crystal data

**Table:**

C_17_H_16_O_5_	*F*(000) = 1264
*M**_r_* = 300.30	*D*_x_ = 1.379 Mg m^−^^3^
Monoclinic, *C*2/*c*	Mo *K*α radiation, λ = 0.71070 Å
Hall symbol: -C 2yc	Cell parameters from 3169 reflections
*a* = 21.063 (15) Å	θ = 3.4–27.5°
*b* = 5.703 (4) Å	µ = 0.10 mm^−^^1^
*c* = 24.437 (18) Å	*T* = 123 K
β = 99.790 (9)°	Rod, colorless
*V* = 2893 (3) Å^3^	0.30 × 0.19 × 0.15 mm
*Z* = 8	

### Data collection

**Table:**

Rigaku/MSC Mercury CCD diffractometer	3297 independent reflections
Radiation source: fine-focus sealed tube	2824 reflections with *I* > 2σ(*I*)
graphite	*R*_int_ = 0.051
ω scans	θ_max_ = 27.5°, θ_min_ = 3.4°
Absorption correction: empirical (using intensity measurements) (*NUMABS*; Higashi, 1999)	*h* = −23→27
*T*_min_ = 0.970, *T*_max_ = 0.985	*k* = −7→5
11426 measured reflections	*l* = −31→31

### Refinement

**Table:**

Refinement on *F*^2^	Primary atom site location: structure-invariant direct methods
Least-squares matrix: full	Secondary atom site location: difference Fourier map
*R*[*F*^2^ > 2σ(*F*^2^)] = 0.084	Hydrogen site location: inferred from neighbouring sites
*wR*(*F*^2^) = 0.146	H-atom parameters constrained
*S* = 1.26	*w* = 1/[σ^2^(*F*_o_^2^) + (0.026*P*)^2^ + 6.5341*P*] where *P* = (*F*_o_^2^ + 2*F*_c_^2^)/3
3297 reflections	(Δ/σ)_max_ < 0.001
201 parameters	Δρ_max_ = 0.29 e Å^−^^3^
0 restraints	Δρ_min_ = −0.28 e Å^−^^3^

### Special details

**Table:**

Geometry. All e.s.d.'s (except the e.s.d. in the dihedral angle between two l.s. planes) are estimated using the full covariance matrix. The cell e.s.d.'s are taken into account individually in the estimation of e.s.d.'s in distances, angles and torsion angles; correlations between e.s.d.'s in cell parameters are only used when they are defined by crystal symmetry. An approximate (isotropic) treatment of cell e.s.d.'s is used for estimating e.s.d.'s involving l.s. planes.
Refinement. Refinement of *F*^2^ against ALL reflections. The weighted *R*-factor *wR* and goodness of fit *S* are based on *F*^2^, conventional *R*-factors *R* are based on *F*, with *F* set to zero for negative *F*^2^. The threshold expression of *F*^2^ > σ(*F*^2^) is used only for calculating *R*-factors(gt) *etc*. and is not relevant to the choice of reflections for refinement. *R*-factors based on *F*^2^ are statistically about twice as large as those based on *F*, and *R*- factors based on ALL data will be even larger.

### Fractional atomic coordinates and isotropic or equivalent isotropic displacement parameters (Å^2^)

**Table:**

	*x*	*y*	*z*	*U*_iso_*/*U*_eq_	
C1	0.12026 (11)	0.2626 (4)	0.39828 (9)	0.0171 (5)	
C2	0.15716 (12)	0.4515 (4)	0.42065 (10)	0.0223 (5)	
H2	0.1686	0.5699	0.3968	0.027*	
C3	0.17771 (13)	0.4698 (4)	0.47766 (10)	0.0229 (5)	
H3	0.2030	0.6000	0.4926	0.027*	
C4	0.16099 (12)	0.2958 (4)	0.51274 (10)	0.0198 (5)	
C5	0.12360 (12)	0.1058 (4)	0.49037 (10)	0.0229 (5)	
H5	0.1120	−0.0124	0.5142	0.027*	
C6	0.10351 (12)	0.0891 (4)	0.43378 (10)	0.0212 (5)	
H6	0.0781	−0.0408	0.4188	0.025*	
C7	0.09682 (11)	0.2342 (4)	0.33803 (10)	0.0176 (5)	
O1	0.06221 (9)	0.0793 (3)	0.31697 (7)	0.0250 (4)	
O2	0.11946 (8)	0.4078 (3)	0.30793 (7)	0.0216 (4)	
C8	0.09736 (11)	0.4149 (4)	0.25035 (9)	0.0181 (5)	
C9	0.06788 (11)	0.6204 (4)	0.22986 (10)	0.0192 (5)	
H9	0.0624	0.7460	0.2542	0.023*	
C10	0.04640 (11)	0.6402 (4)	0.17308 (10)	0.0177 (5)	
H10	0.0254	0.7794	0.1583	0.021*	
C11	0.05562 (11)	0.4570 (4)	0.13782 (10)	0.0169 (5)	
C12	0.08634 (11)	0.2523 (4)	0.15939 (10)	0.0188 (5)	
H12	0.0928	0.1274	0.1352	0.023*	
C13	0.10753 (12)	0.2303 (4)	0.21606 (10)	0.0202 (5)	
H13	0.1286	0.0915	0.2310	0.024*	
C14	0.03310 (11)	0.4762 (4)	0.07712 (10)	0.0177 (5)	
O3	0.00181 (9)	0.6673 (3)	0.06089 (7)	0.0272 (4)	
H3O	−0.0102	0.6630	0.0263	0.041*	
O4	0.04427 (9)	0.3198 (3)	0.04501 (7)	0.0244 (4)	
O5	0.17781 (9)	0.2955 (3)	0.56890 (7)	0.0238 (4)	
C15	0.21608 (12)	0.4869 (4)	0.59482 (10)	0.0202 (5)	
H15A	0.1923	0.6365	0.5875	0.024*	
H15B	0.2568	0.4988	0.5799	0.024*	
C16	0.22997 (12)	0.4371 (4)	0.65666 (10)	0.0207 (5)	
H16A	0.2560	0.2922	0.6636	0.025*	
H16B	0.1889	0.4117	0.6704	0.025*	
C17	0.26641 (13)	0.6408 (5)	0.68816 (11)	0.0277 (6)	
H17A	0.3098	0.6505	0.6788	0.042*	
H17B	0.2696	0.6153	0.7282	0.042*	
H17C	0.2433	0.7875	0.6777	0.042*	

### Atomic displacement parameters (Å^2^)

**Table:**

	*U*^11^	*U*^22^	*U*^33^	*U*^12^	*U*^13^	*U*^23^
C1	0.0169 (12)	0.0177 (11)	0.0174 (12)	0.0001 (9)	0.0053 (9)	−0.0012 (9)
C2	0.0269 (13)	0.0199 (12)	0.0207 (13)	−0.0054 (10)	0.0062 (10)	0.0038 (10)
C3	0.0262 (13)	0.0212 (12)	0.0214 (13)	−0.0056 (10)	0.0042 (10)	0.0002 (10)
C4	0.0189 (12)	0.0255 (13)	0.0156 (12)	0.0005 (10)	0.0044 (10)	0.0003 (10)
C5	0.0275 (14)	0.0237 (12)	0.0184 (13)	−0.0057 (10)	0.0061 (10)	0.0025 (10)
C6	0.0220 (13)	0.0210 (12)	0.0213 (13)	−0.0045 (10)	0.0051 (10)	−0.0004 (10)
C7	0.0181 (12)	0.0163 (11)	0.0192 (12)	0.0005 (9)	0.0058 (10)	0.0002 (9)
O1	0.0298 (10)	0.0258 (9)	0.0203 (9)	−0.0100 (8)	0.0067 (8)	−0.0043 (7)
O2	0.0274 (10)	0.0236 (9)	0.0134 (8)	−0.0069 (7)	0.0024 (7)	0.0006 (7)
C8	0.0174 (12)	0.0232 (12)	0.0138 (12)	−0.0057 (9)	0.0031 (9)	0.0022 (9)
C9	0.0193 (12)	0.0188 (11)	0.0203 (12)	−0.0009 (9)	0.0063 (10)	−0.0032 (9)
C10	0.0175 (12)	0.0176 (11)	0.0184 (12)	0.0006 (9)	0.0045 (9)	0.0013 (9)
C11	0.0136 (11)	0.0193 (11)	0.0181 (12)	−0.0008 (9)	0.0040 (9)	0.0014 (9)
C12	0.0204 (12)	0.0173 (11)	0.0198 (12)	−0.0009 (9)	0.0072 (10)	−0.0012 (9)
C13	0.0208 (12)	0.0199 (12)	0.0200 (12)	−0.0007 (9)	0.0036 (10)	0.0021 (9)
C14	0.0153 (11)	0.0190 (11)	0.0196 (12)	0.0013 (9)	0.0055 (9)	−0.0009 (9)
O3	0.0382 (11)	0.0267 (10)	0.0155 (9)	0.0145 (8)	0.0012 (8)	0.0001 (7)
O4	0.0296 (10)	0.0244 (9)	0.0193 (9)	0.0073 (8)	0.0043 (7)	−0.0031 (7)
O5	0.0281 (10)	0.0259 (9)	0.0168 (9)	−0.0071 (8)	0.0024 (7)	0.0004 (7)
C15	0.0200 (12)	0.0220 (12)	0.0184 (12)	−0.0036 (10)	0.0029 (10)	−0.0007 (10)
C16	0.0181 (12)	0.0269 (13)	0.0174 (12)	0.0002 (10)	0.0035 (10)	0.0009 (10)
C17	0.0284 (14)	0.0338 (15)	0.0200 (13)	−0.0014 (11)	0.0012 (11)	0.0000 (11)

### Geometric parameters (Å, °)

**Table:**

C1—C2	1.385 (3)	C10—H10	0.9500
C1—C6	1.400 (3)	C11—C12	1.394 (3)
C1—C7	1.480 (3)	C11—C14	1.482 (3)
C2—C3	1.391 (4)	C12—C13	1.387 (3)
C2—H2	0.9500	C12—H12	0.9500
C3—C4	1.395 (3)	C13—H13	0.9500
C3—H3	0.9500	C14—O4	1.237 (3)
C4—O5	1.358 (3)	C14—O3	1.300 (3)
C4—C5	1.395 (3)	O3—H3O	0.8400
C5—C6	1.379 (3)	O5—C15	1.438 (3)
C5—H5	0.9500	C15—C16	1.516 (3)
C6—H6	0.9500	C15—H15A	0.9900
C7—O1	1.204 (3)	C15—H15B	0.9900
C7—O2	1.367 (3)	C16—C17	1.527 (4)
O2—C8	1.406 (3)	C16—H16A	0.9900
C8—C9	1.380 (3)	C16—H16B	0.9900
C8—C13	1.385 (3)	C17—H17A	0.9800
C9—C10	1.389 (3)	C17—H17B	0.9800
C9—H9	0.9500	C17—H17C	0.9800
C10—C11	1.389 (3)		
			
C2—C1—C6	119.3 (2)	C10—C11—C14	120.7 (2)
C2—C1—C7	123.2 (2)	C12—C11—C14	119.3 (2)
C6—C1—C7	117.6 (2)	C13—C12—C11	120.2 (2)
C1—C2—C3	120.8 (2)	C13—C12—H12	119.9
C1—C2—H2	119.6	C11—C12—H12	119.9
C3—C2—H2	119.6	C8—C13—C12	118.6 (2)
C2—C3—C4	119.6 (2)	C8—C13—H13	120.7
C2—C3—H3	120.2	C12—C13—H13	120.7
C4—C3—H3	120.2	O4—C14—O3	123.5 (2)
O5—C4—C3	124.9 (2)	O4—C14—C11	121.2 (2)
O5—C4—C5	115.3 (2)	O3—C14—C11	115.3 (2)
C3—C4—C5	119.7 (2)	C14—O3—H3O	109.5
C6—C5—C4	120.2 (2)	C4—O5—C15	118.32 (19)
C6—C5—H5	119.9	O5—C15—C16	107.18 (19)
C4—C5—H5	119.9	O5—C15—H15A	110.3
C5—C6—C1	120.4 (2)	C16—C15—H15A	110.3
C5—C6—H6	119.8	O5—C15—H15B	110.3
C1—C6—H6	119.8	C16—C15—H15B	110.3
O1—C7—O2	122.9 (2)	H15A—C15—H15B	108.5
O1—C7—C1	125.5 (2)	C15—C16—C17	110.8 (2)
O2—C7—C1	111.61 (19)	C15—C16—H16A	109.5
C7—O2—C8	118.23 (18)	C17—C16—H16A	109.5
C9—C8—C13	122.2 (2)	C15—C16—H16B	109.5
C9—C8—O2	116.1 (2)	C17—C16—H16B	109.5
C13—C8—O2	121.7 (2)	H16A—C16—H16B	108.1
C8—C9—C10	118.8 (2)	C16—C17—H17A	109.5
C8—C9—H9	120.6	C16—C17—H17B	109.5
C10—C9—H9	120.6	H17A—C17—H17B	109.5
C11—C10—C9	120.2 (2)	C16—C17—H17C	109.5
C11—C10—H10	119.9	H17A—C17—H17C	109.5
C9—C10—H10	119.9	H17B—C17—H17C	109.5
C10—C11—C12	120.0 (2)		
			
C6—C1—C2—C3	0.2 (4)	C13—C8—C9—C10	1.5 (4)
C7—C1—C2—C3	179.7 (2)	O2—C8—C9—C10	178.5 (2)
C1—C2—C3—C4	0.0 (4)	C8—C9—C10—C11	−1.1 (3)
C2—C3—C4—O5	−179.5 (2)	C9—C10—C11—C12	0.2 (3)
C2—C3—C4—C5	−0.3 (4)	C9—C10—C11—C14	−179.8 (2)
O5—C4—C5—C6	179.7 (2)	C10—C11—C12—C13	0.3 (3)
C3—C4—C5—C6	0.3 (4)	C14—C11—C12—C13	−179.7 (2)
C4—C5—C6—C1	−0.1 (4)	C9—C8—C13—C12	−1.1 (4)
C2—C1—C6—C5	−0.1 (4)	O2—C8—C13—C12	−177.8 (2)
C7—C1—C6—C5	−179.7 (2)	C11—C12—C13—C8	0.2 (4)
C2—C1—C7—O1	−176.3 (2)	C10—C11—C14—O4	175.9 (2)
C6—C1—C7—O1	3.3 (4)	C12—C11—C14—O4	−4.1 (3)
C2—C1—C7—O2	3.8 (3)	C10—C11—C14—O3	−3.7 (3)
C6—C1—C7—O2	−176.6 (2)	C12—C11—C14—O3	176.3 (2)
O1—C7—O2—C8	4.9 (3)	C3—C4—O5—C15	−0.4 (4)
C1—C7—O2—C8	−175.2 (2)	C5—C4—O5—C15	−179.7 (2)
C7—O2—C8—C9	122.6 (2)	C4—O5—C15—C16	−177.7 (2)
C7—O2—C8—C13	−60.5 (3)	O5—C15—C16—C17	−175.9 (2)

### Hydrogen-bond geometry (Å, °)

**Table:**

*D*—H···*A*	*D*—H	H···*A*	*D*···*A*	*D*—H···*A*
O3—H3O···O4^i^	0.84	1.77	2.606 (3)	172.

Symmetry codes: (i) −*x*, −*y*+1, −*z*.
